# Effect of synbiotics on broilers exposed to subclinical doses of fumonisins and deoxynivalenol

**DOI:** 10.1016/j.psj.2025.105809

**Published:** 2025-09-12

**Authors:** Joseph Rishitha Dasireddy, Laharika Kappari, Ramesh K Selvaraj, Todd J. Applegate, Revathi Shanmugasundaram

**Affiliations:** a Department of Poultry Science, University of Georgia, Athens, GA; b Toxicology and Mycotoxin Research Unit, USDA-ARS, Athens, GA

**Keywords:** Mycotoxins, Synbiotic, Growth performance, Cytokines, CD8^+^: CD4^+^

## Abstract

The objective of this study was to identify the efficacy of a synbiotic containing *Bifidobacterium animalis, Pediococcus acidilactici, Enterococcus faecium,* and fructooligosaccharides in mitigating the effects of subclinical doses of combined fumonisins (FUM), deoxynivalenol (DON), and zearalenone (ZEN) in broiler chickens. A total of 360 one-day-old Cobb 500 male chicks were randomly allocated to four treatments in a 2 × 2 factorial design with six replicates per treatment (*n* = 6). The treatments were 1) Control, 2) Mycotoxin-contaminated diet, 3) Synbiotic supplementation (0.05 %, PoultryStar BRO, dsm-firmenich), and 4) Mycotoxin + Synbiotic. The analyzed mycotoxin concentrations in the basal and contaminated diets were 2.4 mg FUM + 1 mg DON + 0.07 mg ZEN and 8.5 mg FUM + 3.8 mg DON + 0.6 mg ZEN per kg of diet, respectively. Data were analyzed by two-way ANOVA. Production performance, intestinal morphology, jejunal tight junction protein expression, splenic and cecal CD4^+^, CD8^+^ T cell percentage, cytokine expression, and oxidative stress markers were evaluated. There were no significant interaction between mycotoxin and synbiotic treatments were observed for production performance. However, on day 35, synbiotic supplementation significantly increased body weight gain (BWG) compared with no synbiotic supplementation groups (*p* < 0.05). Significant interactions between mycotoxin and synbiotic treatments were observed on jejunal crypt depth on d 21, villus length in the jejunum and ileum, occludin mRNA expression, and splenic CD8^+^ T cell percentage and cytokine expression (TNFα and IFNγ) on d 35 (*p* < 0.05). In chickens fed mycotoxin-contaminated diets, synbiotic supplementation increased jejunal crypt depth and ileal villus length, restored CD8^+^ T cell percentages in spleen and cecal tonsils, and downregulated proinflammatory cytokine expression. In conclusion, synbiotic supplementation at 0.05 % alleviated the adverse effects of chronic exposure to subclinical doses of FUM + DON + ZEN by improving growth performance, gut morphology, and immune function in broiler chickens.

## Introduction

Mycotoxins are toxic secondary metabolites produced by various fungal species that contaminate agricultural commodities, particularly feed ingredients. Consumption of mycotoxin-contaminated feed has been associated with significant health issues in humans and productivity losses in livestock (Abdallah et al., 2015). Favorable environmental conditions such as humidity, temperatures, and improper storage often promote the growth of multiple fungi species simultaneously, resulting in co-contamination with more than one mycotoxin (Mohapatra et al., 2017). In North America, major cereal crops are very often contaminated with *Fusarium verticillioides, F. proliferatum,* and *F. graminearum* in the field. As a result, fumonisins (FUM), deoxynivalenol (DON), and zearalenone (ZEN) (Guerre, 2016; Shanmugasundaram et al., 2025) were frequently observed in the feed samples. This contamination leads to substantial economic losses, estimated at $932 million annually in crops and approximately $6 million in livestock, along with $466 million in mitigation costs (Vardon et al., 2003).

Corn is a major poultry feed ingredient, comprising up to 50-70 % of broiler feed formulations in many countries (Guerre, 2016). Regulatory guidelines by the European Union (EU) and Food and Drug Administration (FDA) have established guidance levels for FUM and DON in poultry feed. However, the Annual Corn Mycotoxin Survey reported that corn samples from the United States were frequently contaminated with more than one mycotoxin (dsm-firmenich, 2025). Previous research studies indicate that the co-occurrence of multiple mycotoxins lowers the tolerance level for individual mycotoxins. Additionally, chickens’ susceptibility to FUM and DON depends on the concentration and duration of exposure (Wang and Hogan, 2019). Subclinical concentrations of combined FUM (3-25 mg/kg of feed) and DON (4-4.5 mg/kg of feed) have been shown to reduce growth performance by primarily targeting the gut epithelial barrier, altering the gut microbiota (Paraskeuas et al., 2021), and predisposing the birds to bacterial infections (Danicke et al., 2002; Shanmugasundaram et al., 2022). While FUM inhibits ceramide synthase, altering sphingolipid metabolism and membrane homeostasis (Chen et al., 2021), DON binds to ribosomes and inhibits protein synthesis at the cellular level (Pestka et al., 2004). Consequently, FUM and DON impair intestinal epithelial cell regeneration and immune cell function (Antonissen et al., 2015). This compromised immunological function causes vaccination failure, severity of coccidiosis (Grenier et al., 2016), necrotic enteritis (NE) (Shanmugasundaram et al., 2022), and salmonellosis in chickens (Liu et al., 2020).

Currently available mycotoxin mitigation strategies, such as activated charcoal, organoclays, aluminosilicates, and yeast cell wall components, have limited efficacy against FUM and DON (Kolosova and Stroka, 2011; Murugesan et al., 2015; Vekiru et al., 2007), emphasizing the need for alternative strategies to reduce mycotoxicity. In recent days, microbial supplementation, particularly synbiotics, a combination of probiotics and prebiotics, has gained significant attention in mitigating the adverse impacts of mycotoxins due to their potential to improve broiler growth performance, gut integrity, antioxidant status, and immune function (Min et al., 2016). According to Leary (2007), probiotics lower the gut pH, promote beneficial bacteria, and bind to the mycotoxins to help them be eliminated through feces. Synbiotics may provide broad protection since prebiotics such as fructooligosaccharides (FOS) promote short-chain fatty acid production by selectively increasing beneficial bacteria, especially *Bifidobacteria* and *Lactobacilli,* strengthening epithelial barrier integrity, and modulating immune responses, thereby reducing the immunotoxic effects of mycotoxins (Ashwini et al., 2019). Previous studies have shown that *Lactobacillus acidophilus* (10^10^ CFU) supplementation with prebiotics decreased the adverse effects of DON-induced toxicity (10 mg/kg feed) in weaned piglets (Jeong et al., 2024). *In vitro* studies with human-derived hepatoma cell lines (HepG2) have demonstrated that *Bifidobacterium spp.* can bind zearalenone (90 %), aflatoxin B1 (50 %), and patulin (80 %) (Fuchs et al., 2008). Similarly, *Bifidobacterium* strains isolated from the chicken intestine have been shown to reduce aflatoxin B1 levels *in vitro* (Aalipanah et al., 2022). *In vivo* supplementation with *Enterococcus faecium,* combined with other probiotic strains and mycotoxin binders, has significantly alleviated the adverse effects of 40 μg of AFB1/kg feed in broiler chickens (Guo et al., 2023). *Pediococcus acidilactici NJB421* strain at 2 × 10^8^ CFU mitigated the negative effects of ochratoxin A levels up to 800 μg/kg feed (Das et al., 2021). Similarly, selenium-enriched *P. acidilactici* MRS-7 strain decreased jejunal damage caused by patulin (5 mg /kg) in mice (Chen et al., 2022). In addition, synbiotic supplementation of 20 mg/rat (PoultryStar® Sol) reduced the negative effects of T-2 toxin exposure up to 470 ppb in rats (Assumaidaee et al., 2020).

Despite the demonstrated benefits of probiotics against individual toxins such as AFB1, DON, and T-2 toxins, no study to date has evaluated synbiotic efficacy against combined subclinical exposure to FUM and DON in broilers. Therefore, the objective of this study was to evaluate the efficacy of commercially available synbiotics (PoultryStar® BRO, dsm-firmenich) containing *B. animalis, P. acidilactici, E. faecium,* and FOS in alleviating the negative effects of subclinical exposure to FUM and DON in broilers.

## Materials and methods

### Diets

A non-medicated corn-soybean mealbased mash diet was used as a basal diet and divided into starter (d0-d18) and grower (d19-d35) phases (Table 1). FUM and DON were produced on rice cultures separately using *F. verticillioides* M-3125 and *F. graminearum* DSM-4528, as described earlier (Liu et al., 2023). The homogenized rice cultures were mixed with a basal diet to make experimental diets containing 8.5 mg FUM and 4 mg DON per kg of diet. All treatment diets were contaminated with an average of 0.5 mg of 15-acetyl DON and 3-acetyl DON per kg of feed. Synbiotic (*Bifidobacterium animalis, Pediococcus acidilactici, Enterococcus faecium,* and fructooligosaccharides) at 0.05 % (PoultryStar® BRO, dsm-firmenich) was added to two treatment diets (Synbiotic and Mycotoxin + Synbiotic). LC-MS-MS was used to determine the concentration of mycotoxins in the final diets (Romer Labs, Union, MO, United States; Table 2).

**Table 1:** Ingredient and nutrient composition of the basal diet (as-fed basis).

Ingredient	Starter (%)	Grower (%)
Corn	56.29	64.86
Soybean meal, 48 % CP	37.87	28.44
Soybean oil	2.18	3.80
Dicalcium phosphate	1.48	0.84
Calcium carbonate	0.91	0.78
Sodium chloride	0.40	0.40
MHA	0.37	0.32
L-lysine	0.21	0.22
Trace mineral premix^1^	0.10	0.10
Choline chloride (60 %)	0.07	0.08
L-threonine	0.06	0.07
Vitamin premix^2^	0.05	0.05
Phytase (500FTU)	0.01	0.01

1 Supplied per kilogram of diet: Mn, 107.2 mg; Zn, 85.6 mg; Mg, 21.44 mg; Fe, 21.04; Cu, 3.2 mg; I, 0.8 mg; Se, 0.32 mg.

2 Supplied per kilogram of diet: vitamin A, 5,511 IU; vitamin D3, 1,102 ICU; vitamin E, 11.02 IU; vitamin B12, 0.01 mg; biotin, 0.11 mg; menadione, 1.1 mg; thiamine, 2.21 mg; riboflavin, 4.41 mg; d-pantothenic acid, 11.02 mg; vitamin B6, 2.21 mg; niacin, 44.09 mg; folic acid, 0.55 mg; choline, 191.36 mg.

**Table 2:** Analyzed mycotoxin content of experimental diets.

	Treatment	Total Fumonisins (FUM) (FB1+FB2+FB3) (mg/kg)	FB1(mg/kg)	DON (mg/kg)	ZEN (mg/kg)	Total Mycotoxins (mg/kg)
**STARTER**	Control	2.5	1.8	1.1	0.08	5.6
	Mycotoxin	8.7	6.2	3.8	1.3	18.6
	Synbiotic	2.6	1.9	1.1	0.01	6.3
	Mycotoxin + Synbiotic	8.5	6.3	4.2	1.6	23.0
**GROWER**	Control	2.6	1.9	0.9	0.07	4.7
	Mycotoxin	8.5	6.2	3.8	0.6	16.8
	Synbiotic	2.4	1.8	1.0	0.6	5.0
	Mycotoxin + Synbiotic	8.6	6.2	3.9	0.5	17.0

Representative samples of feeds from treatments were analyzed by HPLC in Romer labs (Union, MO, USA) for fumonisin (FUM), deoxynivalenol (DON), zearalenone (ZEN), and total mycotoxins (including other metabolites like 15 acetyl DON, Type B Trichothecenes, and Aflatoxins) concentrations.8.5 mg/kg FUM and 3.9 mg/kg of DON; 0.05 % synbiotic

### Study design

**Fig. 1:**
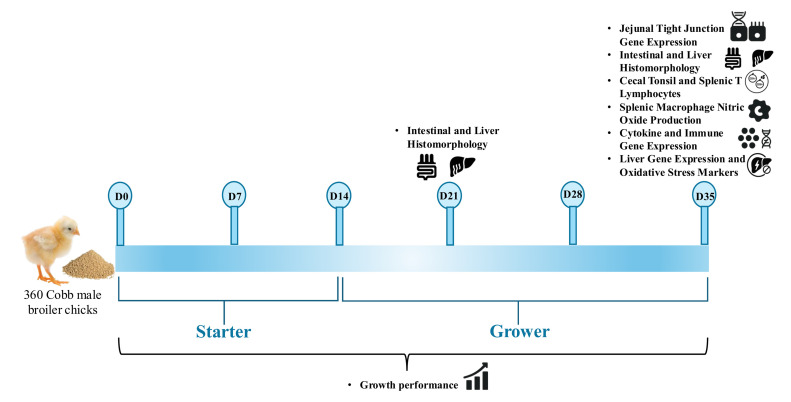
Experimental timeline of the 35-day broiler feeding trial. A total of 360 one-day-old Cobb 500 male broiler chicks were allocated to four dietary treatments (control, mycotoxin, synbiotic, and mycotoxin + synbiotic) in a 2 × 2 factorial arrangement. Growth performance was measured weekly, and sampling was performed at d21 and d35 for intestinal and liver histomorphology, and d35 for jejunal tight junction gene expression, cecal tonsil and splenic T lymphocytes, splenic macrophage nitric oxide production, cytokine and immune gene expression, and liver gene expression with oxidative stress markers.

A 35-day feeding trial was conducted using a total of 360 one-day-old Cobb 500 male broiler chicks (from the Cobb-Vantress Hatchery in Cleveland, GA). Birds were vaccinated at day 0 against *Eimeria* using CocciVAC (Merck Animal Health, NJ, USA). The Institutional Animal Care and Use Committee at the Southern Poultry Research Group, Athens, GA, approved all animal use protocols (AUP: USM102023-117 approved on October 07, 2023). The birds were raised under the supervision of a licensed poultry veterinarian. Day-old broiler chicks were raised in 5 × 5 feet floor pens (stocking density of 1.0 feet^2^ per bird) on new shavings/litter following standard industry practice in the United States. Each pen was provided with one tube feeder and nipple drinker (15 birds to feeder/drinker ratio). The environmental conditions inside the poultry house were controlled by thermostatically controlled heaters and fans. All birds were managed under a standard commercial lighting program, with chicks receiving 23 h of light and 1 h of darkness during the first 2 weeks, followed by 20 h of light and 4 h of darkness beginning in week 3 and continuing through the remainder of the study. Chicks had ad libitum access to feed and water throughout the experimental period. The animal care procedures followed the Guide for the Care and Use of Agricultural Animals in Research and Teaching (McGlone, 2010), and chicks were observed twice daily. Birds were weighed individually and randomly allocated to four treatments with six replicates per group in a 2 × 2 factorial arrangement (n = 6). The experimental treatment groups were Control, Mycotoxin, Synbiotic (Poultry Star BRO, dsm-firmenich), and Mycotoxin + Synbiotic groups. The overall timeline of growth performance measurements and sampling points is shown in Fig. 1.

### Production performance and sampling

Body weight and feed intake were measured at d 0, 7, 14, 21, 28, and 35 of age. Daily mortality was recorded. Average feed intake and BWG were corrected for mortality, and the feed conversion ratio (FCR) was calculated for each pen. On day 35, one bird per pen (n = 6) was euthanized by cervical dislocation for collecting samples. All birds were euthanized using methods approved by the American Veterinary Medical Association (AVMA).

### mRNA transcription analysis

On d35, jejunal mucosal scrapings proximal to Meckel's diverticulum were collected from one bird per pen (n = 6) in RNA later for Mucin-2 (MUC-2) and tight junction proteins like Claudin-1 (CLDN-1), Claudin-2 (CLDN-2), Claudin-4 (CLDN-4), Occludin (OCC), and Zona occludens (ZO) mRNA transcription analysis (Shanmugasundaram et al., 2021). A small piece of spleen and cecal tonsils was collected from each bird per pen (n = 6) for pro-inflammatory [interleukin-1β (IL-1β), interferon-gamma (IFN)γ, tumor necrosis factor-alpha (TNFα)] and anti-inflammatory [interleukin-10 (IL-10) and transforming growth factor-β (TGFβ)] cytokine expression analysis. A small piece of the right caudal liver was collected from one bird per pen (n = 6) in RNA later for quantifying cytochrome P450 (CYP) isoforms CYP1A1, CYP1A2, CYP1A4, CYP3A37, and inducible nitric oxide synthase (iNOS) analysis. After 72 h, the excess *RNA later* was removed from the tissues and stored at -80 °C until further analysis. Total RNA was extracted from the tissues using the phenol-chloroform method and was subjected to spectrophotometry to determine the concentration and quality. A total of 2 μg of RNA was reverse transcribed into cDNA. The cDNA was analyzed for mRNA transcription by real-time PCR (CFX96 Touch Real-Time System, BioRad, Hercules, CA, USA) using SYBR green. The primer sequences and the annealing temperatures are provided in Table 3. The reference genes β-actin, glyceraldehyde-3-phosphate dehydrogenase (GAPDH), and ribosomal protein S13 (RPS13) were selected, and their stability was analyzed using Normfinder software, version 21 (Department of Molecular Medicine, Aarhus University Hospital, Denmark) as described previously (Shanmugasundaram et al., 2018). GAPDH was selected because it exhibited the most stable expression among the reference genes that were tested. Hence, the GAPDH was used for normalizing gene expressions. The relative mRNA expression of the genes was calculated using the 2^−ΔΔCt^ method, where C_t_ is the threshold cycle (Shanmugasundaram et al., 2021). The reference group was the control group without mycotoxin and synbiotic.

**Table 3:** The sequences of primers and real-time quantitative PCR annealing temperatures of the primers used for relative gene expression analysis.

Gene	Primer sequence (5′ - 3′)	Annealing temperature (^°^C)	Accession number
Glyceraldehyde 3-phosphate dehydrogenase (GAPDH)	F-GAGGGTAGTGAAGGCTGCTG R-CCACAACACGGTTGCTGTAT	57.4	NM_001303179.1
Claudin-1 (CLDN-1)	F-CATACTCCTGGGTCTGGTTGGT R-GACAGCCATCCGCATCTTCT	55	NM_001013611.2
Claudin-2 (CLDN-2)	F-CCTGCTCACCCTCATGGAG R-GCTGAACTCACTCTTGGGCT	55	NM_001277622.1
Claudin-4 (CLDN-4)	F-GAAGCGCTGAACCGATACCA R-TGCTTCTGTGCCTCAGTTTCC	57	AY435420.1
Occludin (Occ)	F-GCCTTTTGCTTCATCGCTTCC R-AACAATGATTAAAGCAAAAG	57	NM_205128.1
Zona occludens (ZO)	F-TGTAGCCACAGCAAGAGGTG R-CTGGAATGGCTCCTGTGGT	56	XM_046925212.1
Mucin-2 (MUC-2)	F-ATGCGATGTTAACACAGGACTC R-GTGGAGCACAGCAGACTTTG	59	XM_040673077.2
Interleukin-1β (IL-1β)	F-TCCTCCAGCCAGAAAGTGA R-CAGGCGGTAGAAGATGAAGC	57.5	Y15006.1
Interleukin-10 (IL-10)	F-CATCTCTGGGCCTGAA R-CGTCTCCTTGATCTGCTTGATG	57.5	NM_001004414.4
Transforming Growth Factor β (TGF β)	F-CAGAGCATTGCCAAGAAGC R-GCACGCAGCAGTTCTTCTC	58	HE646744.1
Tumor Necrosis Factor α (TNF α)	F-TTCAGATGAGTTGCCCTTCC R-TCAGAGCATCAACGCAAAAG	57.5	AY765397.1
Interferon γ (IFN γ)	F- CACATATCTGAGGAGCTCTATAC R- GTTCATTCGCGGCTTTG	60	NM_205149.2
Inducible nitric oxide synthase (iNOS)	F- AGTGGTATGCTCTGCCTGCT R- CCAGTCCCATTCTTCTTCC	60	Q90703
Cytochrome P450 1A1 (CYP1A1)	F-AGATCTGGAAGGACCCCTCC R-TAGGAGGCCAGCTGATTCCT	57	NM_205147.2
Cytochrome P450 1A2 (CYP1A2)	F-GCTTTGACACCGTGACAACC R-GTCTGCCTTCTTGCCATCCT	57	NM_205146.3
Cytochrome P450 1A4 (CYP1A4)	F-CAGAACGCCCTGAAGACCTT R-CAAGGCAGCGTACATCATGC	55.4	X99453.1
Cytochrome P 450 3A37 (CYP3A37)	F-AGCCTGCGGTTGTTGTCATG R-CTTCAGCTAATGAGACAGCGTTTC	60	NM_001001751.1

### Histopathological analysis

On d21 and d35, jejunal and ileal samples and a small piece of right caudal liver from one bird per pen (n = 6) were fixed in a 10 % buffered formalin. Samples were processed using a tissue processor, as described earlier (Shanmugasundaram et al., 2023). Briefly, samples were processed at room temperature in a graded series of alcohols (30 min each in 50 %, 70 %, 95 %, and 100 % ethanol). Samples were cleared using Pro-par (Anatech, Battle Creek, MI), followed by paraffin infiltration at 60°C overnight using a tissue processor (Sakura Finetek USA, Inc., Torrance, CA, USA). Then, the samples were embedded in paraffin blocks. The paraffin blocks were cut into 5-μm cross-sections, and 5 cross-sections of samples were mounted on superfrost slides (Thermo Fisher Scientific, Waltham, MA, USA) and stained with hematoxylin and eosin. The ileum, jejunum, and liver cross-sections were viewed under an Olympus BX60 brightfield microscope (Olympus Corp., Tokyo, Japan) and analyzed using CellSens Imaging software, version 4.3.1 (Olympus America, Central Valley, PA, USA) to measure villi length and crypt depth. Ten intact lamina propria villi and crypts per section and a total of 50 villi per sample were analyzed. Similarly, liver samples were observed for histological alterations like hepatocyte infiltration, cell necrosis, vacuolar degeneration, fibrosis, and bile duct proliferation.

### Spleen and cecal tonsil CD8^+^: CD4^+^ ratio

On d35, the effect of FUM and DON on the spleen and cecal tonsil CD4^+^ and CD8^+^ cell percentages was determined by flow cytometry as described previously (Shanmugasundaram et al., 2015). In brief, single-cell suspensions of spleen and cecal tonsils were enriched for mononuclear cells (MNCs) by density centrifugation over Histopaque (1.077 g/mL, Sigma-Aldrich, St. Louis, MO) for 15 min at 400 × g. The cells were incubated with a 1:250 dilution of fluorescent-isothiocyanate-conjugated mouse anti-chicken CD4^+^ (Southern Biotech, Birmingham, AL), a 1:450 dilution of phycoerythrin-conjugated mouse anti-chicken CD8^+^ (Southern Biotech, Birmingham, AL), and a 1:200 dilution of unlabeled mouse IgG for 15 min. The unbound antibodies were removed by centrifugation. The cells were washed three times with wash buffer. The percentages of CD4^+^ and CD8^+^ cells were analyzed using a flow cytometer (Guava EasyCyte, Millipore, MA), and the CD8^+^: CD4^+^ ratio was calculated.

### Nitric oxide assay

On d35, a small piece of spleen was collected from one bird per cage (n = 6) in 5 mL of RPMI-1640, placed on ice, and transported to the laboratory. Single-cell suspension of the spleen was obtained by using a 45 μm cell strainer (Fisher Scientific). Single-cell splenocytes were layered on Histopaque (1.077 g/mL, Sigma-Aldrich, St. Louis, MO), and mononuclear cells were obtained by density centrifugation. The splenocyte MNCs were washed and resuspended in 8 mL of complete RPMI-1640 medium (media supplemented with 4 % FBS, 2 % chicken serum, and 1 % penicillin plus streptomycin), plated in T75 cell culture flasks, and incubated in a 5 % CO_2_ incubator at 40 °C for 24 h. After 24 h of incubation, the adherent cells were removed by trypsinization (5 mL of 0.4 % trypsin + 0.025 % EDTA). Splenic macrophages were then washed in complete media and resuspended in 5 mL of complete RPMI for counting. Splenocyte MNCs were reseeded in 96-well plates (250 μL/well of 1 × 10^5^ cells/mL) in duplicates. Cells were stimulated with 10 μg/mL of *Salmonella* Enteritidis LPS (Sigma Chemicals, MO, USA) and incubated for 48 h. After incubation, plates were centrifuged at 400 × g for 10 min, and the nitrite levels in the supernatant were determined using the Griess method (Green et al., 1982). Nitrite concentration was measured at an absorbance of 540 nm using a Synergy HTX multimode microplate reader (BioTek, VT, USA). The nitrite concentration in the samples was determined using a standard curve of serially diluted sodium nitrite.

### Glutathione assay

A small piece of the right caudal liver was collected and cryopreserved in liquid nitrogen and stored at -80 °C until use. Glutathione (GSH) concentration of liver tissue was measured using a Glutathione Assay kit (Cayman Chemical Item No. 703002) following the manufacturer's instructions. Briefly, the PBS-rinsed liver tissue was homogenized in 5-10 mL of 20 mM HEPES buffer, pH 7.2 (containing 1 mM EGTA, 210 mM mannitol, and 70 mM sucrose per gram of tissue) and centrifuged at 10000 × g for 15 min at 4 °C. The collected supernatant was deproteinated by adding an equal volume of metaphosphoric acid (MPA) reagent (5 g MPA of in 50 mL of water) and then centrifuged at 2000 × g for 2 min. The sample supernatant was mixed with 50 µL of 4 M triethanolamine (531 µL of triethanolamine with 469 µL of water) per 1 mL of supernatant and vortexed immediately. 50 µL of standards and 50 µL of deproteinated samples were added to a 96-well plate. 150 µL of assay cocktail mixture was added to each well, and the plate was incubated for 25 min at room temperature. The OD value was measured at absorbance 405 nm using a Synergy HTX multimode microplate reader (BioTek, VT, USA), and the GSH concentration was presented as a mean OD 405.

### Thiobarbituric acid reactive substances (TBARS) assay

TBARS concentration was measured using the Quantichrom^TM^ TBARS Assay kit (Bioassay Systems, Hayward, CA) following the manufacturer's instructions. Briefly, the serum samples (n = 6) were deproteinized with 200 µL of ice-cold 10 % trichloroacetic acid to 100 µL of each sample and incubated on ice for 5 min. Then, samples were centrifuged at 14000 X g for 5 min, and 200 µL of supernatant was transferred into separate tubes. The colorimetric assay was done with malondialdehyde (MDA) standards and samples on a 96-well plate. Thiobarbituric acid (TBA) reagent was mixed with an equal volume of samples and standards. After vortexing, 100 µL of each sample and standards was plated in a 96-well plate, and the plates were read at the absorbance of 535 nm using a Synergy HTX multimode microplate reader (BioTek, VT, USA). TBARS results are expressed as MDA concentrations in μM.

### Statistical analysis

A two-way ANOVA (JMP Pro 15 software, Cary, NC, USA) was conducted to analyze the effects of feed mycotoxins and synbiotic supplementation on the dependent variables, with the pen being considered as an experimental unit. Data were checked using the Shapiro-Wilk test for normality and Levene's test for homogeneity of variance prior to ANOVA, and assumptions were met. When there were no significant interactions between FUM + DON and synbiotics (*p* > 0.05), the main effects were analyzed, and the effects that had *p* values between 0.05 and 0.1 were mentioned as a trend; when there were significant interactions between treatments, the differences between means were separated by Tukey’s HSD. Values are reported as means ±SEM.

## Results

### Effects of synbiotic supplementation on production performances in broiler chickens fed mycotoxin-contaminated diet

There were no significant interaction effects of mycotoxins and synbiotic supplementation on BWG and FCR at 7, 14, 21, 28, and 35 days of age (Table 4). On d35, there was a significant main effect of synbiotic supplementation on BWG. Among the treatment groups, synbiotic supplementation significantly increased BWG by 6.9 % compared to the no synbiotic treatments (*p* < 0.05).

**Table 4:** Effects of synbiotic supplementation on production performances in broiler chickens fed mycotoxin-contaminated diets.

Treatments	0-7d			0-14d			0-21d			0-28d			0-35d		
	BW (kg)	FI (kg)	FCR	BW (kg)	FI (kg)	FCR	BW (kg)	FI (kg)	FCR	BW (kg)	FI (kg)	FCR	BW (kg)	FI (kg)	FCR
Control	0.090	0.12	1.32	0.358	0.487	1.36	0.762	1.07	1.40	1.479	2.10	1.42	2.208	3.23	1.46
Mycotoxin	0.098	0.14	1.40	0.349	0.477	1.37	0.742	1.04	1.41	1.387	2.00	1.44	2.067	3.07	1.48
Synbiotic	0.098	0.13	1.36	0.369	0.498	1.35	0.792	1.09	1.38	1.500	2.12	1.41	2.294	3.32	1.45
Mycotoxin+ Synbiotic	0.096	0.13	1.32	0.356	0.480	1.35	0.786	1.10	1.39	1.499	2.12	1.42	2.277	3.34	1.46
**Mycotoxin**															
No mycotoxin	0.094	0.13	1.34	0.363	0.492	1.36	0.777	1.08	1.39	1.490	2.11	1.42	2.251	3.28	1.46
Mycotoxin	0.097	0.13	1.36	0.353	0.479	1.36	0.764	1.07	1.40	1.443	2.06	1.43	2.172	3.20	1.48
**Synbiotic**															
No synbiotic	0.094	0.13	1.36	0.354	0.482	1.36	0.752	1.05	1.40	1.433	2.05	1.43	2.138^b^	3.15	1.47
Synbiotic	0.097	0.13	1.34	0.363	0.490	1.35	0.789	1.09	1.39	1.499	2.12	1.41	2.285^a^	3.33	1.46
**SEM**	0.003	0.002	0.047	0.009	0.008	0.023	0.019	0.06	0.024	0.037	0.12	0.016	0.061	0.57	0.01
***p*-value**															
Mycotoxin	0.39	0.52	0.70	0.24	0.28	0.92	0.49	0.11	0.59	0.22	0.15	0.49	0.21	0.26	0.15
Synbiotic	0.39	0.53	0.72	0.32	0.42	0.51	0.06	0.31	0.50	0.09	0.26	0.32	0.03	0.35	0.20
Mycotoxin*Synbiotic	0.12	0.62	0.21	0.81	0.15	0.87	0.70	0.22	0.81	0.24	0.17	0.58	0.33	0.48	0.97

The birds were supplemented with 0.05 % synbiotic from D0 to D35. Mycotoxin-contaminated diets were fed from d0 to d35 in a 2 × 2 factorial setup. Mycotoxin- 8.5mg/kg FUM + 3.9mg/kg DON. Synbiotic- 0.05 %. BWG and mortality-adjusted FCR were recorded weekly to assess the production performance. Mean values (±SEM) in the same row with no common superscript differ significantly. BWG, body weight gain; FCR, feed conversion ratio; SEM, standard error of mean.

### Effects of synbiotic supplementation on jejunal tight junction proteins mRNA transcription levels in broiler chickens fed mycotoxin-contaminated diet

**Fig. 2:**
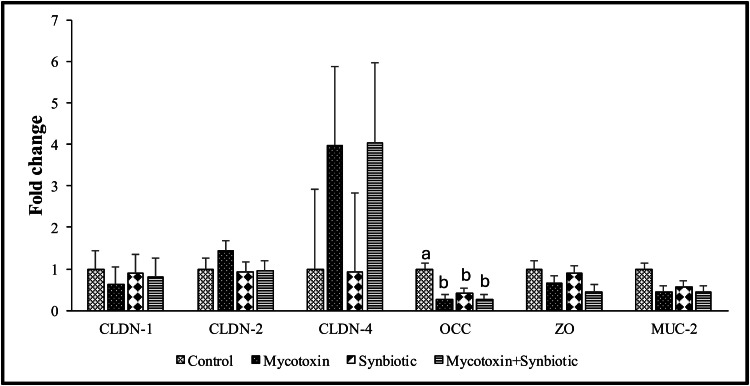
Effects of synbiotic supplementation on jejunal tight junction proteins mRNA transcription levels in broiler chickens fed mycotoxin-contaminated diet. The birds were supplemented with 0.05 % synbiotic from d0 to d35. Mycotoxin-contaminated diets were fed from d0 to d35 in a 2 × 2 factorial setup. Bars (± SEM) with no common superscript differ significantly (p < 0.05). Mycotoxin- 8.5mg/kg FUM + 3.9mg/kg DON. Synbiotic- 0.05 %. On d35, jejunal mucosal scrapings were analyzed for the expression of CLDN-1, CLDN-2, CLDN-4, OCC, ZO, and MUC-2 genes using qPCR. p values: For CLDN, Claudin; OCC, Occludin; ZO, Zona occludens; MUC, Mucin 2 protein.

On d35, there were no significant interaction effects of mycotoxin and synbiotic supplementation on mRNA transcription levels of jejunal CLDN-1, 2, 4, ZO, and MUC-2 genes (*p* > 0.05) (Fig. 2). However, there were significant main effects of mycotoxin on the jejunal ZO and MUC-2 mRNA transcription levels (*p* < 0.05). Mycotoxin exposure significantly downregulated ZO and MUC-2 mRNA transcription levels by 1.8-fold and 2.2-fold, respectively, compared to the no mycotoxin group (*p* < 0.05).

On d35, there were significant interaction effects between mycotoxin and synbiotic supplementation on jejunal OCC mRNA transcription levels between treatment groups (Fig. 2) (*p* < 0.05). Synbiotic supplementation significantly downregulated the jejunal OCC mRNA transcription levels compared to the no synbiotic treatments (*p* < 0.05).

### Effects of synbiotic supplementation on jejunal, ileal and liver histomorphology in broiler chickens fed mycotoxin-contaminated diets

On day 21, there was a significant interaction between mycotoxin and synbiotic supplementation on the jejunal crypt depth (*p* < 0.05), in which synbiotic supplementation significantly increased the jejunal crypt depth by 54.7 % in broilers fed mycotoxin contaminated diet (*p* < 0.05) (Table 5). There were no significant interaction effects of mycotoxin and synbiotic supplementation on jejunal villus length, ileal villus length, or crypt depth. However, there were significant main effects of mycotoxin on the ileal villus length and crypt depth (*p* < 0.05), where mycotoxin exposure decreased ileal villus length and crypt depth by 17.6 % (*p* < 0.05). On d21, a significant main effect of synbiotic supplementation was observed on ileal crypt depth (*p* < 0.05), where synbiotic supplementation significantly increased the crypt depth by 27.8 %, regardless of mycotoxin exposure (*p* < 0.05).

**Table 5:** Effects of synbiotic supplementation on jejunal and ileal villus length and crypt depth in broiler chickens fed mycotoxin-contaminated diets.

Treatments	d21					d35			
	Jejunum		Ileum				Jejunum	Ileum	
	Villus length(μm)	Crypt depth(μm)	Villus length(μm)	Crypt depth(μm)	Villus length(μm)		Crypt depth(μm)	Villus length(μm)	Crypt depth(μm)
Control	898.67	206.83^a^	912.65	196.06	1092.83		229.60	1110.65^a^	207.48
Mycotoxin	767.39	154.88^b^	675.00	156.07	793.88		176.44	738.59^b^	153.72
Synbiotic	902.85	222.40^a^	975.90	243.56	1129.83		227.08	989.42^ab^	194.70
Mycotoxin+Synbiotic	1013.28	239.73^a^	881.14	206.36	1204.45		217.53	1256.01^a^	174.98
**Mycotoxin**									
No mycotoxin	900.76	214.61	944.28^a^	219.81^a^	1111.30		228.34	1050.00	201.09^a^
Mycotoxin	890.34	197.30	778.07^b^	181.22^b^	999.20		196.98	997.30	164.35^b^
**Synbiotic**									
No synbiotic	833.03	180.85^b^	793.83	176.07^b^	943.4^b^		203.02	924.60	180.60
Synbiotic	958.07	231.06^a^	928.52	224.96^a^	1167.1^a^		222.30	1122.70	184.84
**SEM**	73.99	15.04	65.61	14.84	90.15		15.33	97.74	14.51
**p-value**									
Mycotoxin	0.89	0.26	0.02	0.02	0.23		0.05	0.60	0.02
Synbiotic	0.11	< 0.01	0.05	< 0.01	0.02		0.22	0.06	0.77
Mycotoxin*Synbiotic	0.12	0.03	0.29	0.93	0.05		0.17	< 0.01	0.25

The birds were supplemented with 0.05 % synbiotic from d0 to d35. Mycotoxin-contaminated diets were fed from d0 to d35 in a 2 × 2 factorial setup. Mycotoxin- 8.5mg/kg FUM + 3.9mg/kg DON. Synbiotic- 0.05 %. Jejunal and ileal sections were stained with hematoxylin and eosin. Villi height and crypt depth were measured using cellSens Imaging software. Mean values (±SEM) in the same row with no common superscript differ significantly. SEM, standard error of the mean.

On d35, there were significant interaction effects between mycotoxin and synbiotic supplementation on the jejunal and ileal villus length (*p* ≤ 0.05), where synbiotic supplementation in the mycotoxin treatment group increased the jejunal villus length by 51.7 % and the ileal villus length by 70.1 % (*p* < 0.05). There were no significant interaction effects between mycotoxin and synbiotic supplementation on jejunal and ileal crypt depth (*p* < 0.05). There was a significant main effect of mycotoxin on the jejunal and ileal crypt depth (*p* < 0.05), where mycotoxin decreased the jejunal and ileal crypt depth by 13.7 % and 18.2 %, respectively (*p* < 0.05).

On d21 and d35, mycotoxin treatment groups showed increased necrosis and disrupted villus tips in the ileum and jejunum compared to the control group. Supplementing with synbiotics mitigated the lesions induced by the mycotoxin (Supplementary Figures 1 and Figure S2). Similarly, on d21 and d35, histomorphology of liver revealed that hepatocyte degeneration, vacuole, presence of inflammatory cells, hemorrhage, fibrosis and bile duct proliferation in the mycotoxin group. Synbiotic supplementation mitigated the mycotoxin-induced lesions (Figure S3).

### Effects of synbiotic supplementation on cecal tonsils and spleen CD4^+^, CD8^+^ T lymphocytes in broiler chicken fed mycotoxin-contaminated diets

There was a significant interaction between mycotoxin and synbiotic supplementation on the cecal tonsil CD8^+^ T cell percentage (*p* < 0.05), where synbiotic supplementation significantly increased the cecal tonsil CD8^+^ T cell population in the birds fed mycotoxin-contaminated diets and was comparable to the control group (Table 6). There were no significant interaction effects between mycotoxin and synbiotic supplementation on cecal tonsil CD4^+^ T cells percentage and CD8^+^: CD4^+^ ratio. There was a significant main effect of mycotoxin on the CD4^+^ T cell percentage and CD8^+^: CD4^+^ ratio in the cecal tonsils (*p* < 0.05), where mycotoxin increased the CD4^+^ T cell percentage by 33.7 % and decreased the CD8^+^:CD4^+^ ratio by 30.1 % (*p* < 0.05). Also, there was a significant main effect of synbiotic supplementation on cecal tonsil CD8^+^: CD4^+^ ratio (*p* < 0.05), in which synbiotic supplementation increased the CD8^+^: CD4^+^ ratio by 20.1 % compared to the no synbiotic treatment groups.

**Table 6:** Effects of synbiotic supplementation on cecal tonsils and spleen CD4^+^, CD8^+^ T lymphocytes in broiler chicken fed mycotoxin-contaminated diets.

Treatments	Cecal Tonsil			Spleen		
	CD8^+^	CD4^+^	CD8^+^:CD4^+^	CD8^+^	CD4^+^	CD8^+^:CD4^+^
Control	14.42^a^	8.92	1.63	39.60^a^	14.43	2.82
Mycotoxin	11.17^b^	11.07	1.02	29.12^b^	15.45	1.94
Synbiotic	12.82^ab^	7.30	1.80	36.26^ab^	14.22	2.57
Mycotoxin+Synbiotic	14.55^a^	10.62	1.38	37.09^a^	16.27	2.32
**Mycotoxin**						
No mycotoxin	13.62	8.11^b^	1.72^a^	37.93	14.32	2.70^a^
Mycotoxin	12.86	10.84^a^	1.20^b^	33.11	15.86	2.13^b^
**Synbiotic**						
No synbiotic	12.79	9.99	1.33^b^	34.36	14.94	2.38
Synbiotic	13.68	8.96	1.59^a^	36.68	15.24	2.45
**SEM**	0.99	0.73	0.11	2.45	1.34	0.18
**p-value**						
Mycotoxin	0.45	< 0.01	< 0.01	0.06	0.27	< 0.01
Synbiotic	0.38	0.17	0.02	0.36	0.82	0.71
Mycotoxin*Synbiotic	0.02	0.43	0.37	0.03	0.71	0.09

The birds were supplemented with 0.05 % synbiotic from d0 to d35. Mycotoxin-contaminated diets were fed from d0 to d35 in a 2 × 2 factorial setup. Mycotoxin- 8.5mg/kg FUM + 3.9mg/kg DON. Synbiotic- 0.05 %. CD4+, CD8+ T cells, and CD8+/CD4+ ratio in cecal tonsils were analyzed by flow cytometry. Mean values (±SEM) in the same row with no common superscript differ significantly. SEM, standard error of the mean.

In the spleen, there were significant interactions between mycotoxin and synbiotic supplementation on the CD8^+^ T cell population (*p* < 0.05). Mycotoxin significantly decreased the spleen CD8^+^ T cell percentage by 26.5 % (*p* < 0.05) compared to the control group. There were no significant interactions between mycotoxin and synbiotic supplementation on spleen CD4^+^ T cell percentages and CD8^+^: CD4^+^ ratio. However, there was a significant main effect of mycotoxin on the spleen CD8^+^: CD4^+^ ratio (*p* < 0.05), where the mycotoxin decreased the CD8^+^: CD4^+^ ratio by 21.1 % compared to no mycotoxin treatment groups (*p* < 0.05).

### Effects of synbiotic supplementation on splenic macrophage nitric oxide production in broiler chickens fed mycotoxin-contaminated diets

**Fig. 3:**
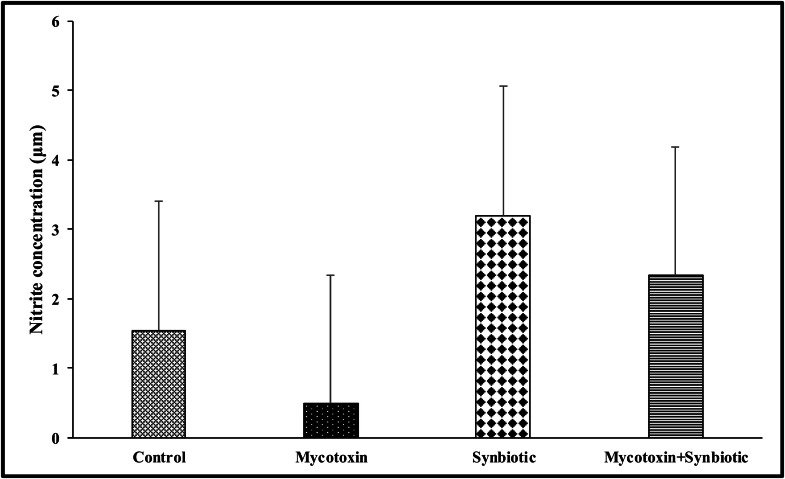
Effects of synbiotic supplementation on splenic macrophage nitric oxide production in broiler chickens fed mycotoxin-contaminated diets. The birds were supplemented with 0.05 % synbiotic from d0 to d35. Mycotoxin-contaminated diets were fed from d0 to d35 in a 2 × 2 factorial setup. Mycotoxin- 8.5mg/kg FUM + 3.9mg/kg DON. Synbiotic- 0.05 %. On d 35, the splenocyte MNCs (1 × 10^5^ cells) were challenged in vitro with LPS (1 mg/mL) for 48 h, and then nitric oxide concentration was measured using the Griess assay. Bars (± SEM) with no common superscript differ significantly (p < 0.05). p values: mycotoxin*synbiotic = 0.84, mycotoxin = 0.05, synbiotic = 0.0012.

On d35, there were no significant interactions between mycotoxin and synbiotic supplementation on the splenic macrophage nitric oxide production, followed by LPS stimulation (Fig. 3). However, there were significant main effects of mycotoxin and synbiotic supplementation on splenic macrophage nitric oxide production (*p* < 0.05). The FUM + DON decreased nitric oxide production by 40.6 %, whereas the synbiotic supplementation increased the nitric oxide production by 173.9 % (*p* < 0.05) compared to the no synbiotic supplementation groups.

### Effects of synbiotic supplementation on cecal tonsils IL-1β, IFNγ, TNFα, IL-10, and TGFβ mRNA transcription levels in broiler chickens fed mycotoxin-contaminated diets

**Fig. 4:**
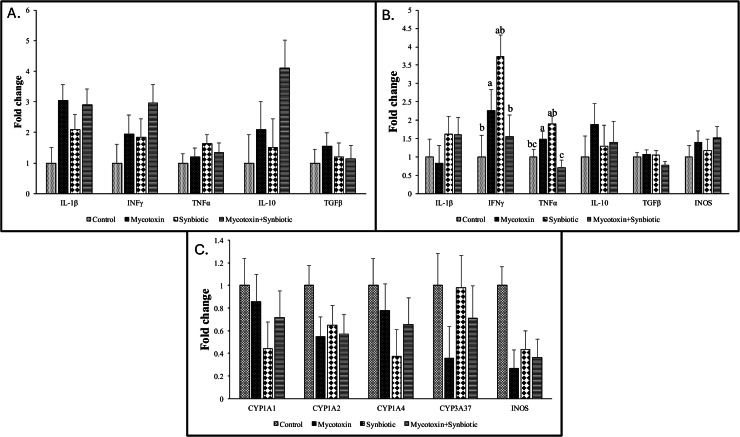
Effects of synbiotic supplementation on cecal tonsils IL-1β, IFNγ, TNFα, IL-10, and TGFβ, spleen IL-1, IFNγ, TNFα, IL-10, TGFβ, and iNOS and liver Cytochrome P450 and iNOS mRNA transcription levels as in broiler chickens fed mycotoxin-contaminated diets. The birds were supplemented with 0.05 % synbiotic from d0 to d35. Mycotoxin-contaminated diets were fed from d0 to d35 in a 2 × 2 factorial setup. Mycotoxin- 8.5mg/kg FUM + 3.9mg/kg DON. Synbiotic- 0.05 %. Fig. 4A: On D35, the cecal tonsils were analyzed for the expression of IL-1β, IL-10, TGFβ, TNF-α, and IFN-γ genes using qPCR. Bars (± SEM) with no common superscript differ significantly (p < 0.05). p values: For IL-1β, Interleukin; TNF, Tumor necrosis factor; TGF, transforming growth factor; IFN, Interferon. . Fig. 4B: On d35, the spleen was analyzed for the expression of IL-1, IL-10, TGFβ, TNFα, INOS, and IFNγ genes using qPCR. Bars (± SEM) with no common superscript differ significantly (p < 0.05). p values: For IL, Interleukin; TNF, Tumor necrosis factor; TGF, transforming growth factor; IFN, Interferon. . Fig. 4C: On d35, the liver was analyzed for the expression of CYP1A1, CYP1A2, CYP1A4, CYP3A37, and iNOS genes using qPCR. Bars (± SEM) with no common superscript differ significantly (p < 0.05). p values: For CYP1A1, CYP1A2, CYP1A4, CYP3A37, iNOS. .

On d35, there were no significant interaction effects between mycotoxin and synbiotic supplementation on cecal IL-1β, TNFα, IFNγ, TGFβ, and IL-10 mRNA transcription levels (Fig. 4A). However, there was a significant main effect of mycotoxin on cecal tonsils IL-1β transcription (*p* < 0.05), and a trend was observed in IL-10 transcription level (*p* = 0.06). The mycotoxin contamination upregulated IL-1β and IL-10 transcription by 3-fold compared to the no mycotoxin group.

### Effects of synbiotic supplementation on spleen IL-1, IFNγ, TNFα, IL-10, TGFβ, and iNOS mRNA transcription levels in broiler chickens fed mycotoxin-contaminated diets

There were significant interactions between mycotoxin and synbiotic supplementation on spleen IFNγ and TNFα mRNA transcription levels (*p* < 0.05) (Fig. 4B). The mycotoxin increased the IFNγ transcription by 2.3-fold, whereas synbiotic supplementation downregulated the IFNγ transcription levels in the mycotoxin-treated group by 1.5-fold (*p* < 0.05) compared to the no mycotoxin and no synbiotic control group. The mycotoxin exposure increased the spleen TNFα transcription levels by 1.5-fold (*p* < 0.05), whereas synbiotic supplementation in the mycotoxin exposed groups downregulated the TNFα expression by 1.4-fold (*p* < 0.05) compared to the no mycotoxin and no synbiotic control group.

There were no significant interactions or main effects between mycotoxin and synbiotic supplementation on spleen IL-1, TGFβ, IL-10, or iNOS mRNA transcription (Fig. 4B).

### Effects of synbiotic supplementation on liver Cytochrome P450 and iNOS mRNA transcription levels in broiler chickens fed mycotoxin-contaminated diets

On d35, there were no significant interaction effects or main effects between mycotoxin and synbiotic supplementation on the liver CYP1A1, CYP1A2, CYP1A4, or CYP3A37 mRNA transcription (Fig. 4C). There was no significant interaction between mycotoxin and synbiotic supplementation on the liver iNOS mRNA transcription levels (*p* > 0.05). However, there was a significant main effect of mycotoxin on the liver iNOS mRNA transcription levels (*p* < 0.05), where the mycotoxin exposure downregulated liver iNOS mRNA transcription levels by 3.2-fold (*p* < 0.05) compared to the no mycotoxin treatment groups.

### Effects of synbiotic supplementation on liver glutathione concentrations in broiler chickens fed mycotoxin-contaminated feed

**Fig. 5:**
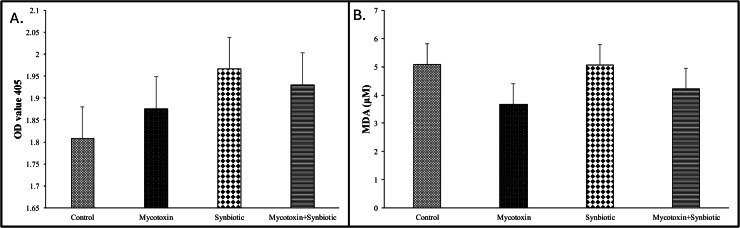
Effects of synbiotic supplementation on liver glutathione concentrations and serum TBARS in broiler chickens fed mycotoxin-contaminated feed. The birds were supplemented with 0.05 % synbiotic from d0 to d35. Mycotoxin-contaminated diets were fed from d0 to d35 in a 2 × 2 factorial setup. Fig. 5A: On D35, the liver tissue was analyzed for the Glutathione activity. Bars (± SEM) with no common superscript differ significantly (p < 0.05). P values: mycotoxin*synbiotic = 0.48, synbiotic = 0.16, mycotoxin = 0.82. Fig. 5B: On D35, the serum was analyzed for the Thiobarbituric acid reactive substances (TBARS) concentration. Bars (± SEM) with no common superscript differ significantly (p < 0.05). p values: mycotoxin*synbiotic = 0.70, synbiotic = 0.72, mycotoxin = 0.14.

On d35, there were no significant interactions or main effects between mycotoxin and synbiotic supplementation on the glutathione concentrations in the liver of broiler chickens between treatment groups (*p* > 0.05) (Fig. 5A).

### Effects of synbiotic supplementation on serum TBARS in broiler chickens fed mycotoxin-contaminated feed

On d35, there were no significant interactions or main effects between mycotoxin and synbiotic supplementation on the serum TBARS concentrations in broiler chickens (Fig. 5B).

## Discussion

Synbiotics have gained significant attention due to their ability to enhance the gut microbiota, improve nutrient absorption, modulate immune responses, and overall act as an alternative to antibiotics in the poultry industry (Song et al., 2022). Previous studies from our laboratory demonstrated that when chickens were exposed to combined doses of 3 mg FUM and 4 mg DON for 35 days, they exhibited decreased production performance and a reduced relative abundance of cecal Lactobacillaceae (Shanmugasundaram et al., 2023). Hence, in this study, we evaluated the efficacy of 0.05 % synbiotic supplementation in mitigating the adverse effects of subclinical concentrations of FUM and DON in broilers. Our results demonstrated that synbiotic supplementation mitigated the mycotoxin induced negative effects on growth performance by increasing villi length-to-crypt depth ratio, modulated the immune response by restoring CD8^+^: CD4^+^ T cells in the spleen and cecal tonsils, increased nitric oxide production in splenic macrophages, and decreased expression of proinflammatory cytokines, TNFα and IFNγ in broiler chickens.

In the present study, on d35 chronic exposure to subclinical doses of combined 8.5 mg FUM + 4.0 mg DON numerically decreased BWG in broiler chickens by 141 grams (6.4 %) and increased the FCR by 2 points compared to the control group. Previous study with combined doses of 3 mg FUM + 4 mg DON per kg diet for 35 days exposure resulted in 84 g reduction in BWG and a 3-points increase in FCR (Shanmugasundaram et al., 2023). Similarly, a combined dose of 20 mg FUM and 5 mg DON per kg diet for 21 days resulted in a 41 g decrease in BWG and a 3-point increase in FCR (Liu et al., 2020). In the present study, on d35 synbiotic supplementation effectively mitigated the FUM + DON induced decrease in body weight. Synbiotic supplementation increased the BWG by 210 grams (10.2 %) and decreased the FCR by 1 point compared to the mycotoxin treatment group. While the exact mechanisms have not been completely demonstrated, i*n vitro* studies using LAB bacteria supplementation in Caco-2 cell lines showed that probiotic cell wall components, such as peptidoglycans, can bind to AFB1, reducing their bioavailability and cytotoxic effects on gut epithelial cells (Gratz, 2007). In addition to probiotic component FOS selectively stimulates beneficial bacteria such as *Bifidobacteria* and *Lactobacilli*, which can improve epithelial barrier integrity. In this present study, a similar mechanism may be involved in the observed increase in BWG following synbiotic supplementation, may counteract the disruption caused by FUM and DON, thereby supporting nutrient absorption. These research findings align with a study on weaned piglets, where dietary DON exposure at 10 mg/kg of DON for 28 days decreased BWG by 19 %, but synbiotic supplementation (1 % of cello-oligosaccharides, 10^10^ colony-forming unit (CFU) of *Lactobacillus acidophilus* + 10^9^ *Devosia insulae*/animal) mitigated the DON-induced reduction in BWG (Jeong et al., 2024).

The small intestine is the primary site for nutrient absorption, and intact microstructural features such as villi length and crypt depth serve as key indicators of gut health (Awad et al., 2008). A higher villus length and crypt depth is associated with enhanced epithelial cell turnover, which is beneficial for nutrient absorption and overall intestinal function (Fan et al., 1997; Van Nevel et al., 2005). In the present study, histopathological analysis of the ileum and jejunum on d35 showed a decreased villus length and crypt depth in the group exposed to mycotoxin. This finding is consistent with the previous findings in broiler chickens fed 3 mg FUM + 4 mg DON per kg diet for 35 days, which showed a significant reduction in both villus length and crypt depth (Shanmugasundaram et al., 2022). Supplementation with synbiotics appeared to mitigate the mycotoxin-induced reduction in ileal and jejunal villus length and crypt depth. This resulting increase in villus length suggests that increased surface area, which could contribute to improved nutrient absorption and growth performance observed in this study. These results were consistent with previous findings reported by Wu et al., 2018, where dietary supplementation of *Lactobacillus plantarum* JM113 restored the villus length and villus-to-crypt ratio in DON (10 mg/kg) exposed birds. Additionally, supplementation with *Enterococcus faecium* DSM 3530 (5 × 10^8^ CFU/kg diet) in combination with prebiotic inulin has been shown to significantly improve the ileal villi length to crypt depth ratio in broilers (Awad et al., 2008). This significant increase in villi length may be linked to the fermentation of substrates like FOS by probiotic microorganisms, promoting the production of SCFAs, which in turn may stimulate the intestinal epithelial cell turnover and overall promote gut health (Kaewarsar et al., 2023).

In this study, chronic exposure to combined mycotoxins altered the CD8^+^: CD4^+^ ratio on d35, suggesting immune dysregulation. The combined FUM + DON significantly decreased the percentage of CD8^+^ T cells and the CD8^+^: CD4^+^ T cells ratio in the spleen and cecal tonsils. These results are consistent with prior studies in which dietary FUM (8-33 mg/kg) and DON (0.4-3 mg/kg) similarly decreased CD8^+^ and CD4^+^ T cell populations in both the spleen and cecal tonsils (Shanmugasundaram et al., 2025). In the current study, synbiotic supplementation showed potential to mitigate the FUM + DON induced immunosuppressive effects by restoring the CD8^+^ T cell percentage and the CD8^+^: CD4^+^ ratio levels comparable to the control group in both spleen and cecal tonsils. The immunomodulatory effect of synbiotics may, in part, be linked to the production of SCFA, which have been shown to inhibit CD4^+^ T cell proliferation in gut-associated lymphoid tissues (Fontenelle and Gilbert, 2012). Further, SCFAs may contribute to immune homeostasis by limiting T cell recruitment into the blood circulation, thereby decreasing the inflammation associated with mycotoxin exposure (Maslowski et al., 2009).

Tight junction (TJ) proteins are critical for maintaining epithelial barrier integrity by sealing the intercellular spaces between enterocytes (Peterson and Artis, 2014). These protein structures include transmembrane proteins such as claudins and occludins, as well as intracellular scaffolding proteins like Zona occludens (Kozieł et al., 2021). In the present study, jejunal OCC expression was significantly downregulated in the mycotoxin treatment group, with mRNA levels decreased by 3.9-fold compared to the control group. These results align with the previous findings in which 7.7 mg FUM+ 0.4 mg DON for 35d downregulated jejunal OCC expression in broilers (Shanmugasundaram et al., 2025). *In vitro* studies showed that DON (30μmol/L) activates the extracellular-signal-regulated kinases (ERK) signaling pathway, thereby inhibiting TJ protein expression in pig intestinal epithelial cells (IPEC-1) (Pinton et al., 2010). In the present study, synbiotic supplementation did not significantly restore OCC expression in the mycotoxin-exposed birds. However, OCCc transcription is regulated in a complex manner and does not always directly reflect gut barrier integrity. Reduction in OCC expression can be compensated by changes in other TJPs, such as ZO and CLDN proteins, without compromising gut epithelial barrier function (White et al., 2024). In our experiment, the decrease in OCC expression coincided with improved villus morphology and increased body weight, suggesting that synbiotic supplementation did not impair, but rather supported the gut epithelial barrier function. Similar results were reported in broilers supplemented with a synbiotic (*E. faecium, P. acidilactici, B. animalis, L. reuteri*, and FOS), where no significant differences were observed in ileal ZO and CLDN-1 (White et al., 2024). It is important to understand that some mycotoxin metabolites retain toxic properties (Nahle et al., 2022). For instance, the reduced form of ZEN retains estrogenic activity (Hahn et al., 2015), and aflatoxicol, a reduced derivative of AFB1, retains its DNA binding potential (Karabulut et al., 2014). While this study did not quantify FUM or DON metabolites directly, the altered expression of TJ proteins may be partly ascribed to increased SCFA production by synbiotics, particularly butyrate. *In vitro* studies with Caco-2 cells have shown that supplementation with 5.0 mM butyrate can differentially regulate the TJ proteins, significantly increasing OCC expression by 115 %, while decreasing CLDN-1 and CLDN-2 by 39-90 % (Valenzano et al., 2015). Despite no significant interaction effects between mycotoxins and synbiotic supplementation on jejunal CLDN-1, CLDN-2, CLDN-4, ZO, or MUC-2, mycotoxin exposure alone significantly downregulated jejunal ZO and MUC-2 gene expression. These findings are in agreement with previous research in which diets containing 22.9 mg FUM + 4.3 mg DON per kg exhibited decreased MUC-2 expression and altered mucin monosaccharide profiles (Antonissen et al., 2015).

Macrophage nitric oxide acts as a cytotoxic effector molecule, plays a pivotal role in macrophage-mediated host defense against pathogenic bacteria, viruses, fungi, and parasites. In macrophages, NO production is predominantly catalyzed by iNOS, which is upregulated by cytokines, particularly IFNγ and TNFα (Den Haan and Kraal, 2012); (Korhonen et al., 2005). In the current study, exposure to combined mycotoxins significantly decreased NO production following LPS stimulation. These findings are in agreement with previous findings showing that dietary exposure to 14 mg/kg FUM and 3.5 mg/kg DON decreased splenic iNOS expression and NO production in chickens (Shanmugasundaram et al., 2025). Similar immunosuppressive effects have been reported in murine macrophages co-cultured with FUM and DON, where LPS-induced NO production was significantly decreased (Moon, 1998; Sugiyama et al., 2011). In this present study, synbiotic supplementation in the mycotoxin group restored NO production in splenic macrophages following LPS stimulation. This immunomodulatory effect is most likely associated with the synergistic action of FOS and structural components of probiotic microorganisms, such as lipoteichoic acids and peptidoglycans, which interact with pattern recognition receptors, and can promote iNOS expression. An *in vitro* study using murine macrophage cell lines demonstrated that *Bifidobacterium* strains increased NO production by upregulating iNOS-dependent stimulation via mitogen-activated protein kinase (MAPK) and nuclear factor kappa B (NF-κB) signaling pathways (Zabłocka et al., 2024).

Cytokines are important signaling molecules that regulate the immune response (Libby, 2007). In the current study, proinflammatory cytokines, IFNγ, and TNFα in the spleen were significantly upregulated in the mycotoxin treatment group compared to the control group. These findings were consistent with a previous study in which rats exposed to FB1 at 25mg/kg for 8 h exhibited increased hepatic expression of TNFα and IL-1β (Bhandari and Sharma, 2002). Similarly, DON has been reported to enhance pro-inflammatory cytokine expression, such as TNFα and IL-6, in macrophages via activation of the MAPK/NF-κB signaling pathway (Chung et al., 2003; Jia et al., 2004). In the current study, synbiotic supplementation in the mycotoxin treated groups significantly decreased the spleen TNFα and IFNγ expression by 1.4-fold and 1.5-fold, respectively, compared to the no mycotoxin no synbiotic control group, suggesting that synbiotics exert anti-inflammatory effects, thereby reducing the immunotoxic effects of mycotoxins. Similar results have been reported with *L. plantarum* B1 supplementation decreasing ileal-TNFα expression and mitigating inflammation in broilers challenged with pathogenic *E.coli* (Wang et al., 2017). In the present study, no significant differences were observed in the expression of liver cytochrome P450 enzymes (CYP1A1, CYP1A2, CYP1A4, and CYP3A37) among treatment groups, suggesting minimal involvement of hepatic detoxification pathways or limited absorption of FUM and DON in this model.

Oxidative stress and lipid peroxidation are known adverse effects of FUM and DON at the cellular level (Kövesi et al., 2024; Wang et al., 2016). The activity of the glutathione redox system varies depending on the degree of oxidative stress in the body (Kulcsár et al., 2021). In the present study, there were no significant differences observed in liver GSH levels among the treatment groups. These results are consistent with the previous findings showing no alterations in hepatic glutathione peroxidase activity in broilers exposed to either 10 mg/kg of DON-contaminated diets for 17 days or a combination of 20 mg/kg FUM and 5 mg/kg DON for 39 days (Frankič et al., 2006; Paraskeuas et al., 2021). *In vitro* studies with Caco-2 cell lines have demonstrated that combined exposure to FUM B1 (10 μM) and DON (10 μM) induces a synergistic increase in malondialdehyde (MDA) production, suggesting increased lipid peroxidation compared to single mycotoxin exposure (Kouadio et al., 2007). Probiotic supplementation has previously been shown to mitigate oxidative damage; for instance, supplementation of lactic acid bacteria (LAB) *L. acidophilus* (ACCC11073), *L. plantarum* (CICC21863), and *E. faecium* (CICC20430) restored liver GSH levels in broilers challenged with aflatoxin B1 at 40 µg/kg of feed (Liu et al., 2017). However, in the present study, MDA levels did not differ significantly between treatment groups, suggesting that NADPH availability for GSH synthesis may have remained unaltered (Heshmati et al., 2018); as a result, free radical scavenger systems required for initiating lipid peroxidation may not have been substantially affected (Matthews et al., 2007).

In summary, 0.05 % synbiotic supplementation ameliorated the FUM+DON-induced impairments in growth performance, intestinal morphology, immune regulation, and oxidative status. Synbiotic supplemented birds exhibited increased body weight gain, jejunal and ileal villus length, jejunal occludin expression, improved CD8+: CD4+ T cell ratio in the spleen and cecal tonsil, increased macrophage nitric oxide production, and decreased splenic expression of proinflammatory cytokines TNFα and IFNγ compared to the FUM+DON- treatment groups. It is important to note that in this study, neither FUM, DON, nor their metabolites were quantified in the biological samples, nor were key biomarkers such as the sphinganine to sphingosine ratio assessed. Thus, interpretations involving potential binding or detoxification pathways remain unverified within the scope of this study. Future research should include direct quantification of parent mycotoxins and their metabolites, relative abundance of beneficial bacteria in the different parts of the chicken intestine, toxicity assays of intermediate products of FUM and DON, and identifying potential active regions of probiotic enzymes involved in mycotoxin biotransformation would further help to understand the biological mechanisms by which synbiotics mitigate mycotoxin toxicity.

## Funding

This work was supported by the CRADA with dsm-firmenich (Agreement No. 58-6040-3-002) and 10.13039/100000199USDA ARS award No. 6040-42000-046-000D to RS.

## Disclosures

None of the authors have any conflict of interest

## CRediT authorship contribution statement

**Joseph Rishitha Dasireddy:** Writing – original draft, Methodology. **Laharika Kappari:** Methodology. **Ramesh K Selvaraj:** Writing – review & editing, Methodology. **Todd J. Applegate:** Writing – review & editing, Conceptualization. **Revathi Shanmugasundaram:** Writing – review & editing, Validation, Supervision, Resources, Investigation, Funding acquisition, Conceptualization.

## Appendix Supplementary materials

Image, Image 1

Image, Image 2

Image, Image 3

## Footnotes

Supplementary material associated with this article can be found, in the online version, at doi:10.1016/j.psj.2025.105809.
