# Supplementary figures and images for: Effect of synbiotics on broilers exposed to subclinical doses of fumonisins and deoxynivalenol
Source: Poult Sci. 2025 Sep 12;104(12):105809. doi: 10.1016/j.psj.2025.105809 (PMC12593598; doi:10.1016/j.psj.2025.105809)

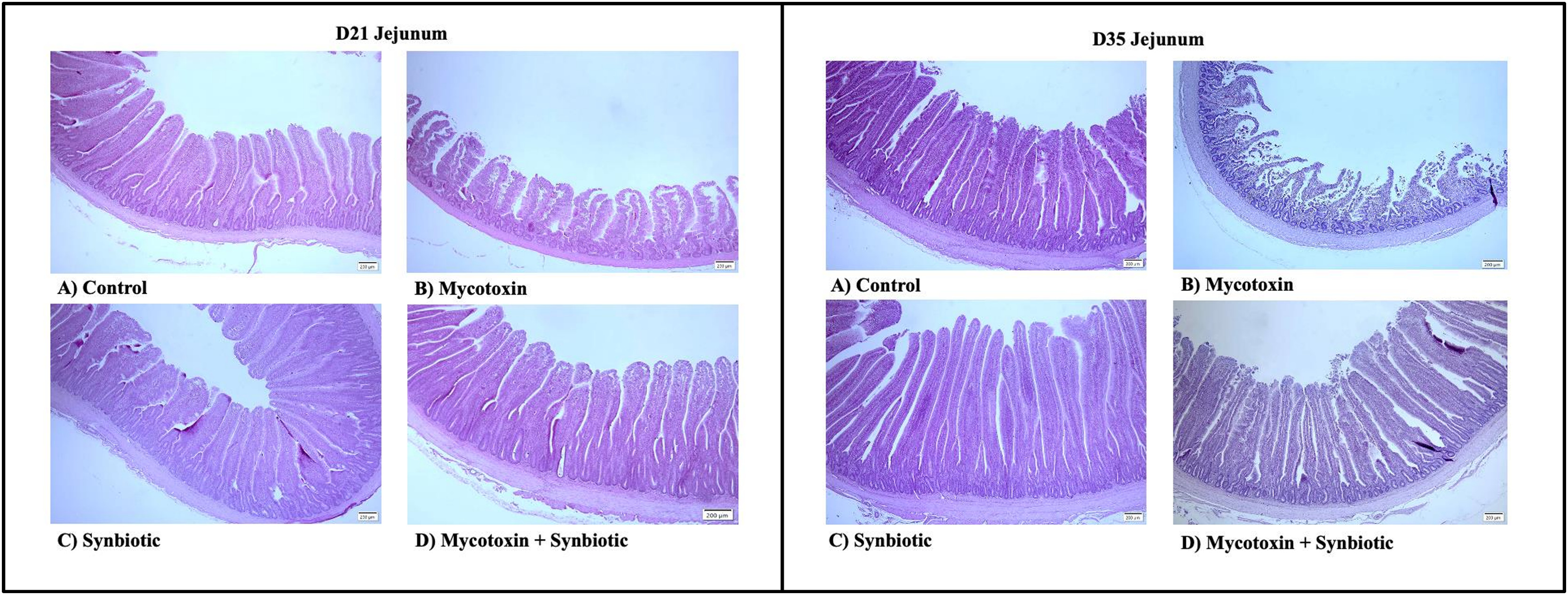

Supplement: Supplementary file 1 [file mmc1.jpg]

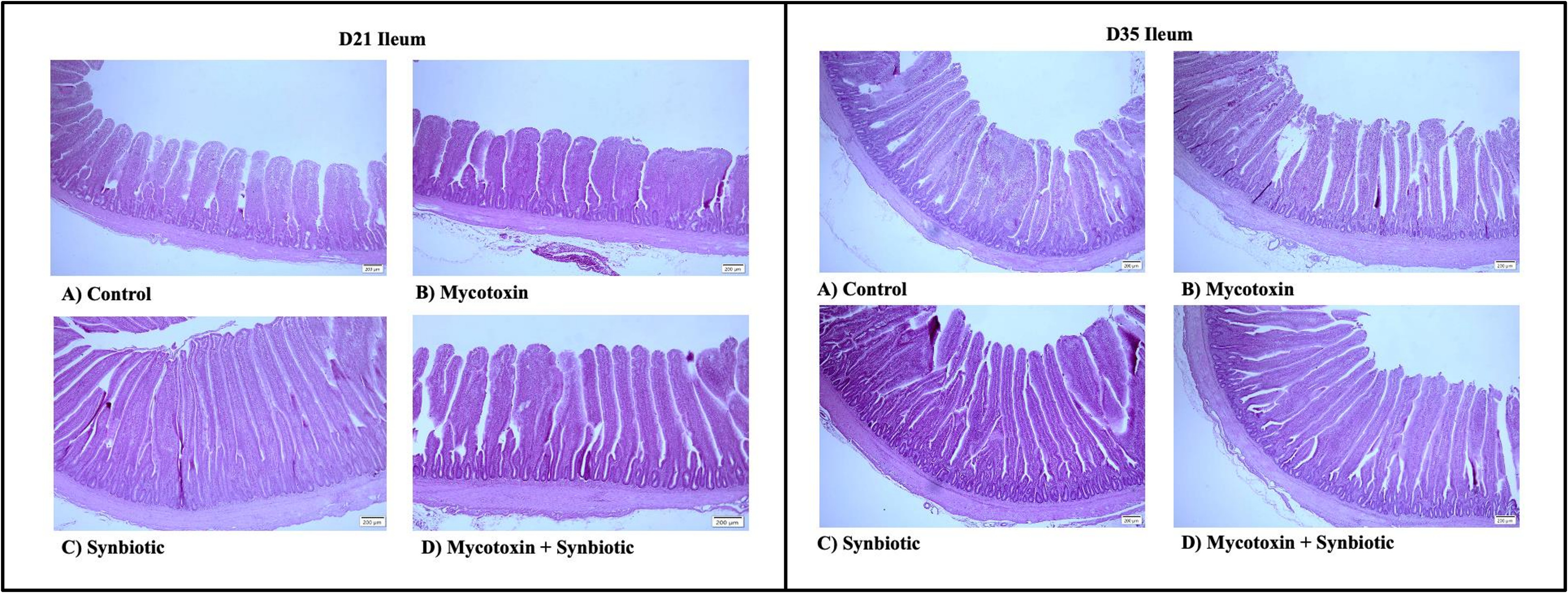

Supplement: Supplementary file 2 [file mmc2.jpg]

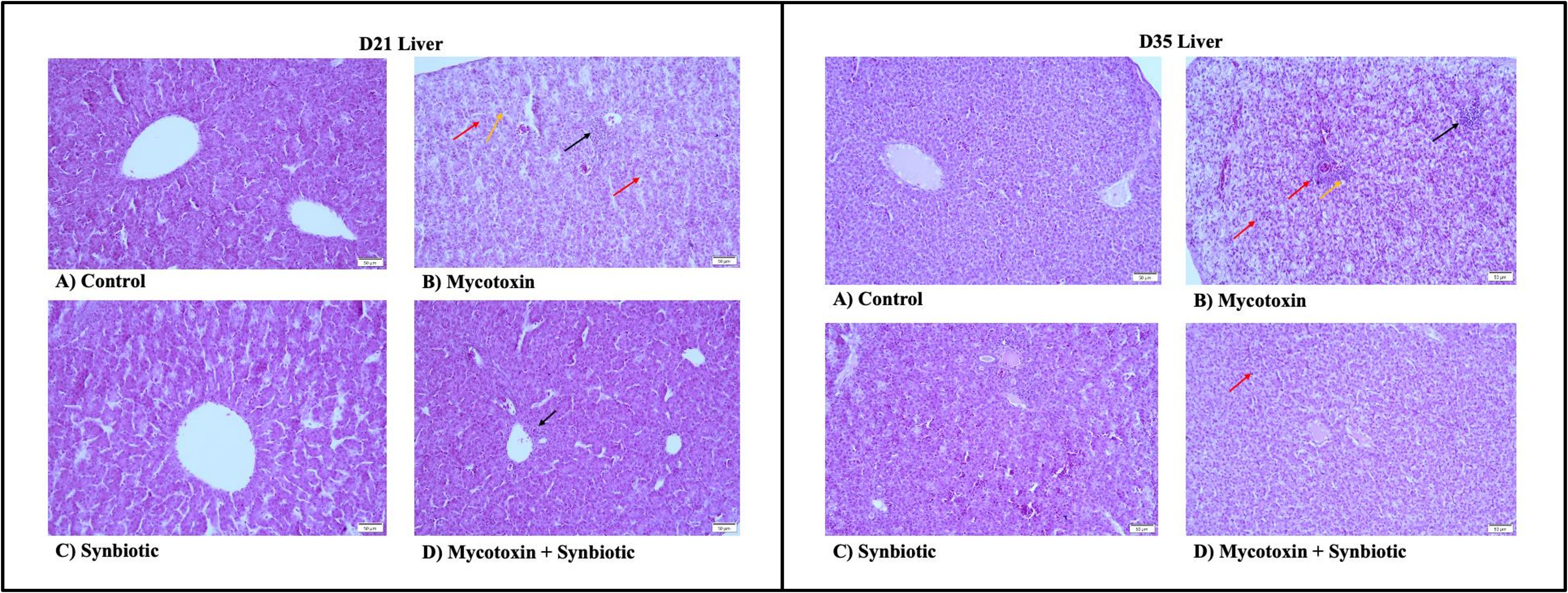

Supplement: Supplementary file 3 [file mmc3.jpg]
